# Alternative Management of Impacted Permanent Central Incisor

**DOI:** 10.5005/jp-journals-10005-1570

**Published:** 2018

**Authors:** Jeswin Thomas, Anoop Harris, Sundeep Hedge, Gen Morgan, Esai A Prabha, Rinu

**Affiliations:** 1,2,4-6 Department of Pedodontics and Preventive Dentistry, Rajas Dental College and Hospital, Chennai, Tamil Nadu, India; 3 Department of Pedodontics and Preventive Dentistry, Yenapoya Dental College, Karnataka, India

**Keywords:** Impacted permanent central incisor, Supernumerary tooth, Treatment

## Abstract

**Aim:**

To discuss about the possible treatment modalities of supernumerary tooth in place of a impacted permanent tooth

**Background:**

Numerical anomaly with an excessive number of teeth are termed as supernumerary teeth.

**Case description:**

This paper describes a case with erupted supernumerary tooth in the place of missing left maxillary central incisor. Radiographically the left maxillary central incisor was impacted below the root of the supernumerary tooth with an additional impacted supernumerary tooth in relation to the right maxillary central incisor.

**Conclusion:**

A multidisciplinary approach is required in managing the supernumerary teeth. When orthodontic extrusion of the impacted permanent tooth is not possible the possibilities of retaining the supernumerary tooth should be considered.

**Clinical significance:**

This paper describes about the treatment of supernumerary tooth erupted in the place of missing left maxillary central incisor not amenable for orthodontic extrusion.

**How to cite this article:**

Thomas J, Harris A, Hedge S, Morgan G, Prabha EA, Rinu. Alternative Management of Impacted Permanent Central Incisor. Int J Clin Pediatr Dent, 2018;11(6):529-531.

## BACKGROUND

Numerical anomaly with an excessive number of teeth is termed as supernumerary teeth. Supernumerary teeth are classified morphologically as rudimentary and supplementary teeth. They are usually associated with different syndromes. They may also appear as an isolated finding in patients with no pathology. They are prevalent in ranges from 0.5–3.8% in permanent teeth and 0.35–0.6% in primary dentition.^1^

Single supernumerary teeth are most commonly noted in anterior maxilla and are associated with the permanent dentition.^2^ The supernumerary teeth occurring between or just posterior to the central incisors are termed as “mesiodens”. They may or may not erupt, and if erupted, it causes malalignment of teeth; if impacted, it has to be evaluated radiologically.^3^

## CASE DESCRIPTION

**Fig. 1:**
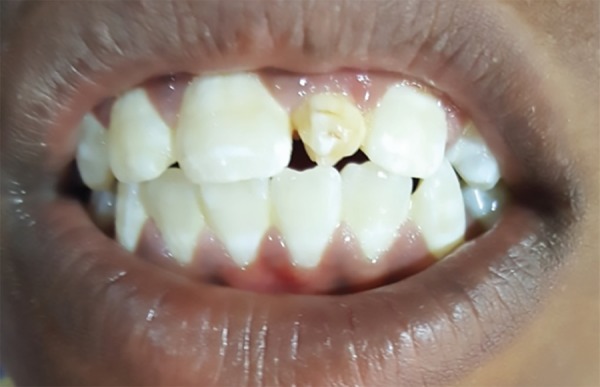
Preoperative

**Fig. 2:**
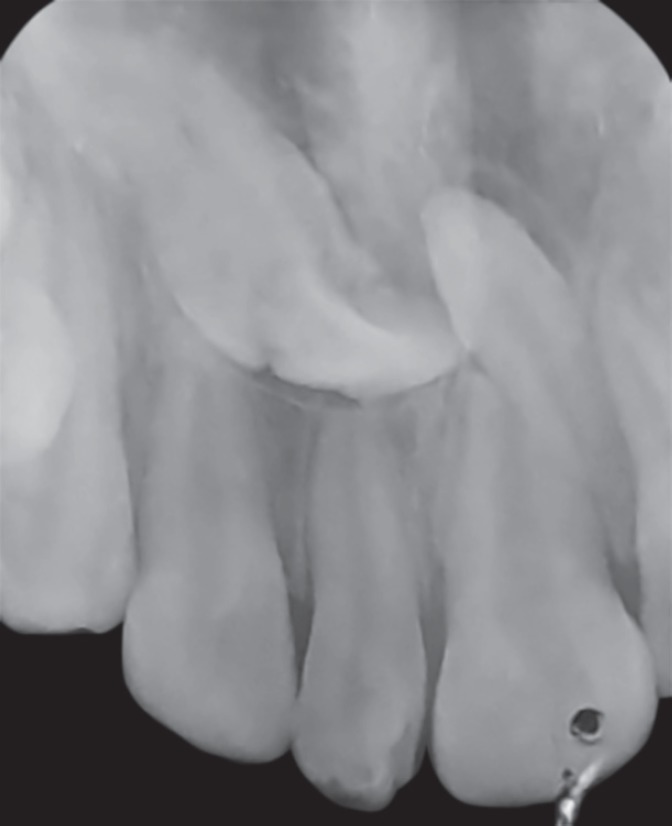
Preoperative IOPA

A 12-year-old male patient reported to the department of pediatric and preventive dentistry, Rajas Dental College and Hospital with a chief complaint of unesthetic appearance. Intraoral examination revealed a cone-shaped supernumerary tooth present in the place of left maxillary central incisor (Fig. 1). The left maxillary central incisor was clinically absent. The supernumerary tooth was caries free, responded to thermal stimuli (heated gutta-percha) and electrical pulp testing. Periodontal probing revealed healthy gingiva and no abnormal mobility was noted. IOPA (Fig. 2) in relation to the supernumerary tooth revealed impacted permanent left maxillary central incisor above the root of the supernumerary tooth. An additional supernumerary tooth was found impacted above the roots of the permanent right maxillary central incisor. No pathosis was seen with the impacted teeth, and no radicular changes were noted.

**Fig. 3:**
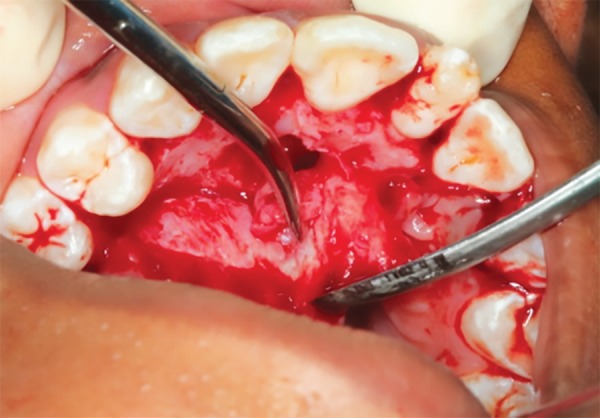
Surgical removal of impacted tooth

**Fig. 4:**
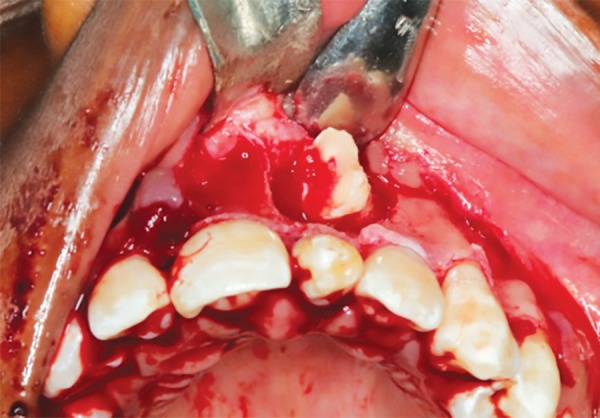
Surgical removal of impacted tooth

**Fig. 5:**
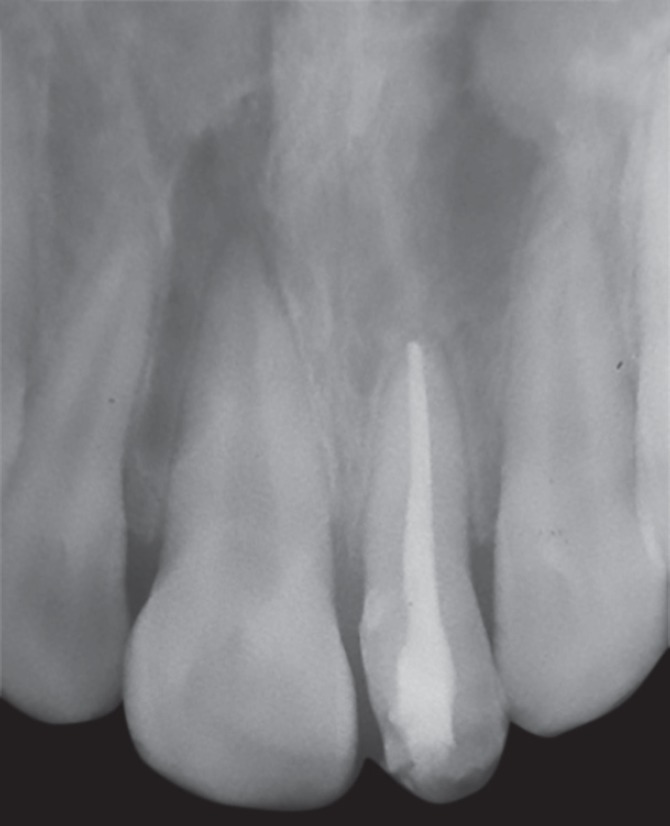
Sutures in place

**Fig. 6:**
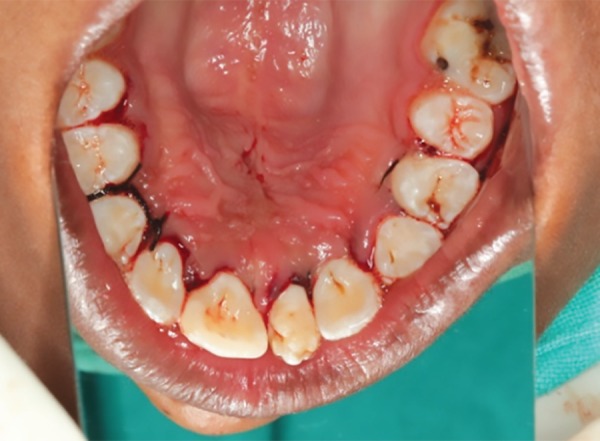
Root canal treated supernumerary tooth

**Fig. 7:**
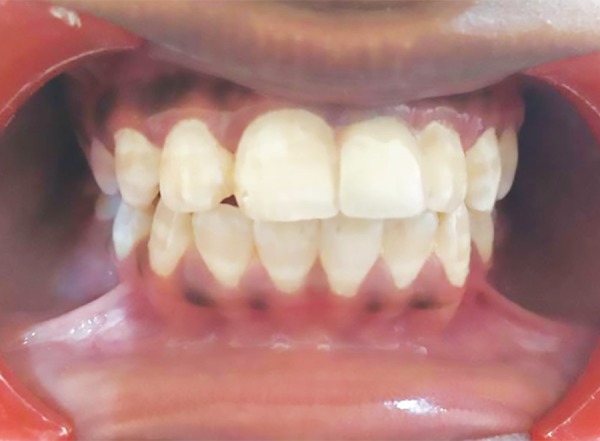
PFM crown

Since the permanent left maxillary central incisor was above the root of the supernumerary tooth, it was decided to retain the supernumerary tooth and surgically remove the left maxillary central incisor and the additional supernumerary tooth. The permanent left maxillary central incisor and the additional supernumerary tooth were removed surgically, and sutures were placed (Figs 3 to 5). Root canal treatment was performed in the supernumerary tooth (Fig. 6). The crown preparation was done and Porcelain-fused-to-metal crowns (PFM's) crown luted (Fig. 7).

## DISCUSSION

The supernumerary tooth is formed from geminated tooth bud or due to the super production of the dental lamina. The conical or tubercular supernumerary tooth do not erupt usually. They may also cause impaction of the associated tooth or retard the eruption of the associated tooth.^1^ Maxillary central incisors impaction is mentioned in the literature.^4^ The impacted central incisor usually erupts after the extraction of the supernumerary tooth, provided they have the eruptive force, and there is sufficient place in the arch.^5^

In this case, the patient's age was taken into consideration (12-year-old), and the radiograph revealed that root of permanent left maxillary central incisor was already completed, this would make spontaneous eruption difficult. Moreover, the tooth was placed in a horizontal position, above the root of the supernumerary tooth making orthodontic repositioning of the tooth difficult. Hence, it was decided to retain the supernumerary tooth since it was free from caries and periodontally sound.

## CONCLUSION

A multidisciplinary approach is required in managing the supernumerary teeth. When orthodontic extrusion of the impacted permanent tooth is not possible, the possibilities of retaining the supernumerary tooth should be considered.

## CLINICAL SIGNIFICANCE

This paper describes the treatment of supernumerary tooth erupted in the place of missing left maxillary central incisor not amenable for orthodontic extrusion.
